# Effectiveness of fluoride-containing toothpastes associated with different technologies to remineralize enamel after pH cycling: an in vitro study

**DOI:** 10.1186/s12903-022-02429-2

**Published:** 2022-11-14

**Authors:** Nayanna Lana Soares Fernandes, José Gabriel Victor Costa Silva, Elizabeth Barreto Galvão de Sousa, Paulo Henrique Perlatti D’Alpino, Andressa Feitosa Bezerra de Oliveira, Elbert de Josselin de Jong, Fábio Correia Sampaio

**Affiliations:** 1 Stricto Sensu Post-Graduate Program in Dentistry, Department of Clinical and Community Dentistry, Health Science Center, Federal University of Paraiba (UFPB), João Pessoa, PB Brazil; 2 Postgraduate Program in Dentistry, Federal University of Minas Gerais, Belo Horizonte, MG Brazil; 3 DentalSchool, Federal University of Paraiba, João Pessoa, PB Brazil; 4 Triplet Biotechnology Solution, São Paulo, SP Brazil; 5 POSMAT – Post-Graduate Program in Materials Science and Technology, School of Sciences, São Paulo State University (UNESP), Bauru, SP Brazil; 6 Department of Morphology, Health Science Center, Federal University of Paraiba, João Pessoa, PB Brazil; 7 Research & Development, Inspektor Research Systems BV, Amsterdam, The Netherlands

**Keywords:** Tooth remineralization, Toothpaste, Dental caries, Hardness, Microscopy, Biomimetics

## Abstract

**Background:**

To evaluate the efficacy of fluoride-containing toothpastes with different technologies to remineralize artificial caries lesions in enamel.

**Methods:**

Bovine enamel blocks were divided into three thirds: intact (untreated), demineralized (artificial caries lesion), and treated (caries lesion, pH cycling with dentifrices). Enamel blocks were randomly distributed into five groups (n = 12): Fluoride-free toothpaste, Colgate Oral Care (NC); Arginine-containing toothpaste, Colgate Total Daily Repair (PC); Silicate-based fluoride toothpaste: REFIX technology, regenerador + sensitive (RDC), NR-5 technology, Regenerate Enamel Science (RES), and NOVAMIN technology, Sensodyne Repair and Protect (SRP). The specimens were submitted to a pH cycling model for 6 days. The efficacy of the toothpastes was estimated by calculating the surface microhardness recovery (%SMH_R_) and the fluorescence recovery (ΔF_RE_) with quantitative light-induced fluorescence. The cross-sectional micromorphology of the enamel surface was also assessed using scanning electron microscopy. Elemental analyses (weight%) were determined with an energy-dispersive X-ray spectrometer (EDS). The results were compared to that of the control (NC). Data were statistically analyzed (5%).

**Results:**

%SMH_R_ could be ranked as follows: RDC = PC = RES = SRP > NC. Significantly higher %SMH_R_ and ΔF_RE_ means were observed after enamel treatment with RDC (22.7 and 46.9, respectively). PC (%SMH_R_ = 18.8) was as efficacious as RDC to recover the surface microhardness with a significantly lower mean of ΔF_RE_ (19.5). Only RDC was able to promote the formation of a mineralized layer on the surface of enamel enriched with silicon on the surface.

**Conclusions:**

The silicate-based fluoride toothpaste containing REFIX technology demonstrated greater efficacy in the remineralizing artificial caries than the other products.

## Background

Tooth brushing with fluoride toothpaste is claimed to significantly reduce the risk of caries associated with sugar intake [1]. Many fluoridated products are available on the market, with different compositions and applications, and are widely used due to their easy access and low cost [2, 3]. Brushing with fluoridated toothpastes is the most effective non-professional intervention to prevent tooth decay, especially in places where the water is not fluoridated [4, 5].

Currently, manufacturers are seeking for innovative compounds to improve the remineralization of dental tissues, with or without fluoride [6–8]. The innovative aspects aim not only to boost the remineralization process but also to increase the regeneration potential of these formulations [7]. Along these lines, these biomimetic agents function as fluoride and calcium carriers for tooth enamel [8]. Biological apatite has been found to be composed of small crystals and characterized by poor crystallinity and relatively high solubility [9]. These alternative mechanisms seem to mimic the natural remineralization, promoting the formation of less soluble and porous hydroxyapatite [10, 11].

The remineralization process can be enhanced by the substituting of ionic species in the sites of the hydroxyapatite molecule [12]. The presence of substitute ions, either incorporated within the apatite lattice or only adsorbed on the surface, including both anionic(e.g., F^−^, Cl^−^, SiO_4_^4−^, and CO_3_^2−^) and cationic substitutions (e.g., Na^+^, Mg^2+^, K^+^, Sr^2+^, Zn^2+^, Ba^2+^, Al^3+^), changes the hydroxyapatite solubility, depending on the substitution at the different sites of the hydroxyapatite molecule (calcium, phosphate, and hydroxyl sites) [9, 13].

Silica, a component of bioactive glass, has been incorporated in some toothpaste formulations in order to enhance their bioactivity and apatite-forming ability of hydroxyapatite [14, 15]. In this manner, silica acts as a site for the precipitation of calcium and phosphate ions, forming calcium silicate, which leads to the nucleation of hydroxyapatite and mineral formation and intensifies the remineralization process [16, 17]. Calcium silicate is responsible for a protective effect on the surface, stimulating the deposition of other minerals and reducing the effects of demineralization [17]. Although it is claimed that these changes seem to occur mainly at the hydroxyapatite surface [18], it seems to be the reason for retaining fluoride in the composition, even at lower concentrations [19].

Therefore, in view of the current efforts in search of effective components against caries, the purpose of this in vitro study was to analyze the efficacy of fluoride-containing toothpastes containing REFIX technology, NR-5 and NOVAMIN technologies to remineralize enamel after pH cycling. The research hypothesis was that the remineralization of the enamel would be positively affected by the use of fluoride toothpastes after pH cycling, irrespective of the technology contained in the products tested.

## Methods

### Sample preparation

The recently extracted bovine incisor teeth were obtained from the commercial establishment: Honorato e Araújo LTDA Fridge (CNPJ: 01.179.091/0001-37) and stored in a 0.08% thymol solution until use. Enamel blocks (4 × 4 × 2 mm) were made from these teeth and then were embedded in self-curing acrylic resin using circular molds of 16 mm in diameter and 3 mm in depth. The outer enamel surface was ground flat with grit papers (600–1500 grades) under water refrigeration and polished with 1 µm diamond paste (Extec Corporation, Enfield, CT) in a rotating polishing machine PSK-2 V (Skill-tec Comércio e ManutençãoLtda, São Paulo, SP, Brazil). Baseline enamel Vickers surface microhardness (SH_0_) analysis was performed with a microhardness tester (Shimadzu HMV—AD Easy Test Version 3.0). Five indentations spaced 100 µm from each other were made at the center of the enamel surface (50 g, 10 s). Enamel blocks between 360 and 400 VHN surface microhardness were selected for the study. Sixty enamel blocks were randomly distributed into 5 groups (n = 12) according to the products used [5, 20, 21].

### Tested groups and interventions

Toothpastes were selected among commercial products containing fluoride associated with different technologies, as indicated by the manufacturers. The characteristics of the products are listed in Table 1. A fluoride-free product was chosen as the control toothpaste (NC) and an arginine-containing toothpaste, Colgate® Total Daily Repair, as the positive control (PC).

**Table 1:** Composition of the toothpastes selected for the study

Product	Active agents	Manufacturer
Fluoride-free toothpaste Colgate oral care (NC)	No active ingredients	Colgate-Palmolive Manufacturing, São Bernardo do Campo, SP, Brazil
Colgate total daily repair (PC)	1450 ppm F-of as sodium fluoride, 0.30% triclosan, arginine, tetrasodium pyrophosphate	Colgate-Palmolive Manufacturing, São Bernardo do Campo, SP, Brazil
Regenerador + sensitive (RDC)	1450 ppm F-of sodium fluoride and tetrasodium pyrophosphate (Refix technology)	Rabbit Corp. Londrina/PR, Brazil
Regenerate Enamel science (RES)	1450 ppm F-of sodium fluoride and sodium monofluorophosphate, calcium silicate and sodium phosphate (NR-5 technology)	Unilever UK Limited, Leatherhead, Surrey, UK
Sensodyne repair and protect (SRP)	1426 ppm of sodium fluoride and calcium sodium phosphosilicate 5% (NOVAMIN technology)	GSK Consumer Healthcare, Norreys Drive, Maidenhead, Berkshire, SL6 4BL, UK

Manufactures’ information

### Lesion formation

Subsurface enamel demineralization was carried out using a modified model [22]. Following 5 min sonication in water using an ultrasonic device, one-third of the exposed enamel surface was covered with two layers of nail varnish (Risqué, Niasi, Taboão da Serra, São Paulo, Brazil) as a reference sound area. The enamel blocks were immersed individually in 32 mL of a demineralizing solution containing 1.3 mM/L Ca(NO_3_)_2_·4H_2_O, 0.78 mM/L NaH_2_PO_4_·H_2_O in 0.05 M/L acetate buffer, 0.03 μgF/mL (NaF), pH 5.0, 32 mL/specimen, during 16 h at 37 °C. After that, the blocks were submitted to a post-demineralization surface hardness (SH_1_) with the same parameters described previously.

### Remineralizing pH-cycling

Before the remineralization pH cycling model [23], the enamel specimens had another one third of its surface covered with two layers of nail varnish (Risqué, Niasi, Taboão da Serra, São Paulo, Brazil) as a reference for caries lesion area. The blocks were submitted to a pH cycling model at 37 °C for 6 days. The blocks were immersed individually in a remineralization solution (1.5 mM L^−1^ calcium, 0.9 mM L^−^1 phosphate, 150 mM L^−1^ potassium chloride in 0.02 mM L^−1^cacodylic buffer, pH 7.0; 0.02 µgF/mL, 1 mL/mm^2^), for 22 h. The cariogenic challenge was performed by a demineralization solution (2.0 mM L^−1^ calcium and phosphate in 75 mM L^−1^ acetate buffer, pH 4.7; 0.03 µgF/mL, 3 mL/mm^2^) during 2 h per day (12–2 pm). Twice a day, at 10 am and 2 pm, enamel samples were exposed to toothpaste slurries (toothpaste: deionized water, 1:3 w/w; 2 mL/enamel specimen) for 1 min, under agitation. Deionized water rinses were performed between each step. In between treatments, the enamel blocks were individually immersed in a remineralization solution at 37 °C. De- and remineralizing solutions were changed daily.

### Microhardness test analysis

The baseline surface microhardness (SH_0_) was evaluated using a microhardness tester with the previously described settings. The enamel surface microhardness after enamel demineralization (SH_1_) and after treatment with the toothpastes associated with pH cycling (SH_2_) was also determined using the same parameters. For this determination, the acid-resistant nail varnish was removed, and the hardness of each third of each specimen was tested. Each indentation in the center of the thirds was separated from the others by a distance of 100 μm. The percentage of surface hardness recovery (%SMH_R_) was then calculated [5, 24, 25], as follows (Eq. 1):

1 $$\% SMHR = \frac{{\left( {SH2 - SH1} \right)}}{{\left( {SH1 - SH0} \right)}} \times 100$$

### Quantitative light-induced fluorescence (QLF) analysis

The bovine enamel blocks were evaluated for fluorescence loss in caries lesions and treated areas, using the Qraycam Pro device (Inspektor Research System BV, Amsterdam, The Netherlands). The nail varnish in each window was carefully removed with a surgical blade and cotton swabs soaked in diluted acetone. Then, the specimens were water rinsed with deionized water and dried with a cotton roll. A camera was attached to a stand in the same position for all the images to standardize the QLF measurements. The images were taken in a dark room, with an exposure of 0, a contrast of 0, and a distance between the device and a sample of 8 cm [26, 27]. A software (Q-ray version 1.38, (Inspektor Research System BV, Amsterdam, The Netherlands) analyzed the changes in the amount of mineral in the enamel based on the ΔF value. The ΔF value represents the percentage decrease in the autofluorescence intensity in a carious lesion and treated areas compared with that of sound enamel, reflecting the changes in the mineral contents of enamel [26]. The measurements were made in two stages for the calculation of the percentage fluorescence recovery (ΔF_RE_): ΔF_0_, which represents a loss of initial fluorescence, passing through the difference between the sound and demineralized enamel, and ΔF_1_, which represents a difference in final fluorescence, using the difference between the sound enamel and the area treated with the toothpastes[28, 29]. Then, the percentage of fluorescence recovery was calculated as follows[29] (Eq. 2):

2 $$\Delta FRE = \frac{{\left( {\Delta F1 - \Delta F0} \right)}}{\Delta F0} \times 100$$

### Scanning electronic microscopy (SEM) plus energy-dispersive X-ray spectroscopy (EDS)

The morphological analysis of the specimens was performed in a scanning electron microscope (EGA 3, TESCAN, LMU, Kohoutovice, Czech Republic), operating at 15 kV. For the morphological analysis of the specimens, the blocks were previously sputter-coated with gold in a vacuum evaporator (MED 010; Balzers, Balzer, Liechtenstein), and then microscopically analyzed to obtain photomicrographs of the surface morphology of the treated specimens (1000× magnification). Representative images of selected regions of the sputter-coated specimens were taken in order to characterize the morphological aspect of the surface [5, 20, 21]. The EDS point analysis (80 mm^2^, SDD Detector, Oxford Instruments, Concord, MA, USA) was performed to determine a qualitative elemental analysis of specimens, operating in high vacuum mode and an accelerating voltage of 15 kV. For each sample, five points were randomly selected for each sample (300 µm^2^ for each point), and the mean values were calculated [20, 21].

For the subsurface analysis, cross-sections of the bovine blocks were obtained by longitudinally sectioning the specimens under water-cooling. Both half-blocks were used for the SEM and the elemental analyses. The halves were dehydrated in silica gel for 3 h. The specimens were then gold-sputtered and evaluated using an SEM coupled with an EDS [20, 21].

### Statistical analysis

Data were analyzed statistically using the SPSS package for Windows, version 21.0 (SPSS, Inc., Chicago, IL, USA). The Shapiro–Wilk test and Levene’s test were used to determine the normality and homogeneity of variances, respectively. As the data demonstrated equal variances and Gaussian distribution, no data transformation was needed. The following tests were performed: (1) ANOVA followed Tukey for the analysis of differences between groups regarding SH_0_, SH_1_, SH_2_, %SMH_R_, and ΔF_RE_; (2) ANOVA repeated measures, followed by Bonferroni, for the analysis of the variables SH_0_, SH_1_, SH_2_ into the same group at the different analysis times; (3) Pearson's correlation between variables. The level of significance considered was 5% [5, 21].

## Results

The average means and standard deviation of surface microhardness for the variables SH_0_, SH_1_, and SH_2_ are displayed in Table 2. In the mineralized surface, the SH_0_ means (baseline enamel surface microhardness) varied from 373.5 (NC) to 384.4 VHN (RDC), with no significant differences between the groups (p > 0.05). No significance was also observed when the SH_1_ means (post-demineralization surface hardness) were compared, varying from 31.4 (RES) to 32.8 VHN (RDC) (p > 0.05). For the variable SH_2_ (surface hardness after pH cycling), the highest mean found was 112.5 (RDC) and the lowest was 38.1 (NC). Significantly higher means of SH_2_ were observed when the enamel was treated with fluoride-containing toothpastes, regardless of the different technology, compared to the control fluoride-free toothpaste (p < 0.05).

**Table 2:** Mean values (standard deviation) of surface hardness analysis according to the different treatments

Product	SH_0_	SH_1_	SH_2_
NC	373.5 (9.4)^a,A^	31.9 (5.4)^b,B^	38.1 (3.7)^c,A^
PC	376.6 (15.6)^a,A^	31.5(4.0)^b,B^	96.5 (11.9)^c,B^
RDC	384.4 (14.3)^a,A^	32.8 (4.4)^b,B^	112.5 (27.3)^c,B^
RES	382.2 (16.5)^a,A^	31.4 (2.6)^b,B^	89.5 (11.7)^c,B^
SRP	377.0 (13.8)^a,A^	32.6 (4.1)^b,B^	81.8 (4.3)^c,B^

Means followed by distinct letters, lower case for rows, upper cases for columns: significant, p < 0.05

SH_0_, surface hardness (baseline); SH_1_, post-demineralization surface hardness; SH_2_, surface hardness after pH cycling; NC, fluoride-free toothpaste colgate; PC, Colgate total 12; RDC, regenerador + sensitive; RES, regenerate enamel science; SRP, sensodyne repair an protect

Table 3 displays the results of QLF analysis for tested areas, ΔF_0_ (sound vs. demineralized area) and ΔF_1_ (sound × treated area) in the experimental groups. No significance was observed for ΔF_0_ when the means were compared (p > 0.05). Conversely, RDC exhibited a significantly higher mean of ΔF_1_ (− 13.5) than all experimental groups (p < 0.05). PC, RES, and SRP presented intermediary means (− 24.1, − 21.3, and − 29.5, respectively). The control group (NC) exhibited a significantly lower mean (− 34.8), than the experimental groups.

**Table 3:** Mean values (standard deviation) of QLF for tested areas, ΔF_0_ (sound *vs.*demineralized area) and ΔF_1_ (soundXtreated area) in the experimentalgroups

Product	ΔF_0_	ΔF_1_
NC	− 27.8 (2.3)^a^	− 34.8 (2.2)^C^
PC	− 29.9 (2.4)^a^	− 24.1 (2.3)^B^
RDC	− 25.4 (7.8)^a^	− 13.5 (5.6)^A^
RES	− 26.4 (9.8)^a^	− 21.3 (11.8)^B^
SRP	− 29.8 (6.3)^a^	− 29.5 (6.6)^B^

Means followed by distinct letters, lower case for ΔF_0_, upper cases for ΔF_1_: significant, p < 0.05

NC, Fluoride-free toothpaste Colgate; PC, Colgate total 12; RDC, regenerador + sensitive; RES, regenerate enamel science; SRP, sensodyne repair and protect

**Fig. 1:**
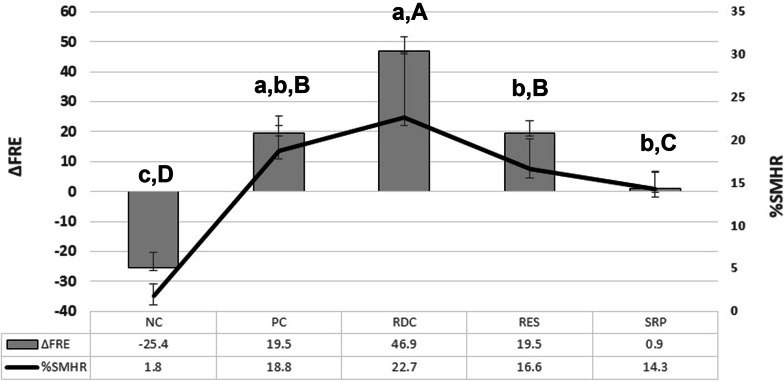
Means and standard deviation of remineralization in terms of fluorescence recovery (ΔF_RE_) and microhardness measurements (%SMH_R_) in the experimental groups. Distinct letters, lower case for %SMH_R_, upper case for ΔF_RE_: significant, p < 0.05. Vertical bars =  ± 1 standard deviation. Abbreviations: NC: Fluoride-free toothpaste Colgate; PC: Colgate Total 12; RDC: Regenerador + Sensitive; RES: regenerate enamel science; SRP: sensodyne repair and protect

In general, the surface microhardness recovery (%SMH_R_) could be ranked as follows: RDC = PC = RES = SRP > NC. When the %SMH_R_ results were compared, RDC and PC exhibited significantly higher means, followed by RES and SRP (p < 0.05)(Fig. 1). After treatment with the fluoride-free toothpaste, a significantly lower mean of %SMH_R_ was observed (p < 0.05). RDC exhibited the higher mean ΔF_RE_, being statistically different from all groups, followed by PC and RES. SRP exhibited the lowest ΔF_RE_ (0.27) of the fluoride-containing toothpastes (Fig. 1). The fluoride-free toothpaste (NC) exhibited a negative mean of ΔF_RE_ (− 6.63). The statistical analysis demonstrated that significantly higher means of %SMH_R_ and ΔF_RE_ were observed after enamel treatment with RDC (22.7 and 46.9, respectively). The anti-erosive, arginine-containing toothpaste (PC) was as effective as RDC to recover the surface microhardness (%SMH_R_ = 18.8), but comparatively exhibited a significantly lower mean of ΔF_RE_ (19.5). A strong positive correlation was found when variables ΔF_RE_ and %SMH_R_ were plotted (r^2^ = 0.9371, p < 0.001).

**Fig. 2:**
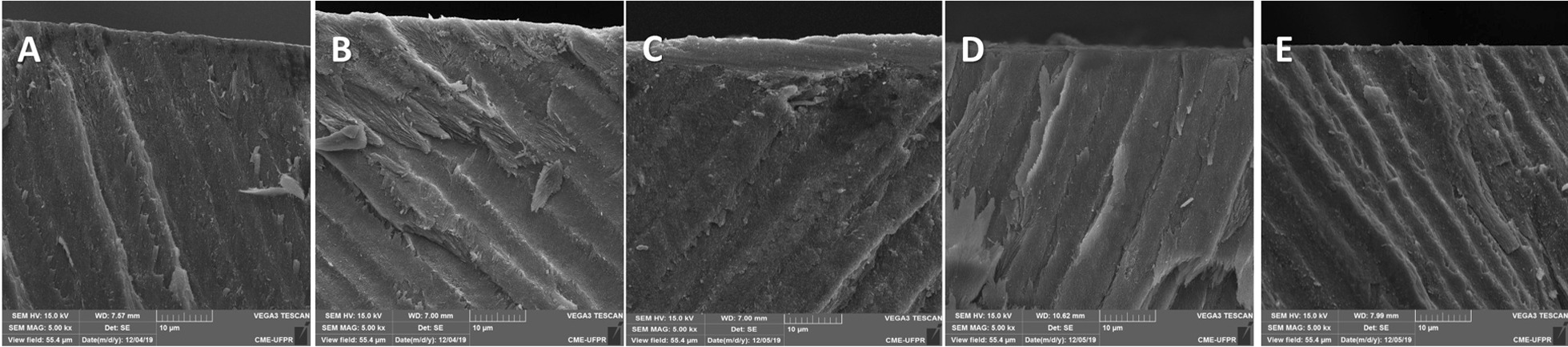
Representative scanning electron micrographs of the enamel cross-sections. **a** NC; **b** PC; **c** RDC; **d **RES; **e** SRP. Abbreviations: NC: Fluoride-free toothpaste Colgate; PC: Colgate total 12; RDC: regenerador + sensitive; RES: regenerate enamel science; SRP: sensodyne repair and protect

Figure 2 shows representative scanning electron micrographs of the enamel cross-sections and demonstrates the differences among the experimental groups. RDC induced the formation of a mineralized layer onto the enamel surface when associated with pH cycling. This mineralized surface layer was not observed when the enamel blocks were treated with other toothpastes. Table 4 shows the elemental mapping analysis was shown demonstrating the differences among the groups. RDC exhibited the highest silicon content (7.14%). Variability in the Ca/P ratio was observed in the analyzed specimens can also be seen in Table 4, with the lowest ratio for PC (1.58) and the highest for RES (1.96). EDS also showed that the element fluoride was more frequent in surfaces treated with SRP and PC and less present in RDC and RES.

**Table 4:** Elemental mapping according to the experimental groups

Element	NC	PC	RDC	RES	SRP
C	49.51	8.8	54.83	28.71	16.78
O	38.79	27.54	32.36	36.22	35.45
F	0.00	0.41	0.05	0.05	1.13
Na	0.20	0.69	0.10	0.32	0.41
Mg	0.00	0.51	0.15	0.29	0.34
Al	0.00	0.00	0.25	0.00	0.00
Si	0.29	0.00	7.14	0.07	0.00
K	0.00	0.00	0.19	0.00	0.00
Cl	0.15	0.53	0.00	0.41	0.00
Ca/P	1.75	1.58	1.86	1.96	1.94

NC, Fluoride-free toothpaste Colgate; PC, Colgate total 12; RDC, regenerador + sensitive; RES, regenerate enamel science; SRP, sensodyne repair and protect

## Discussion

The results of the present study demonstrated different values for enamel caries recovery among the analyzed toothpastes, as related in other studies [30–36]. Although no significance was observed when comparing the results of the microhardness after pH cycling (SH_2_), irrespective of the treatment technology, RDC and PC exhibited significantly higher means of microhardness, followed by RES and SRP (Fig. 1.). The calculus of %SMH_R_, which considers the microhardness in the different testing areas, demonstrated a significantly higher mean when the enamel was treated with RDC compared to other experimental groups.

QLF is a visible light system used to quantitatively and nondestructively monitor the progression or regression of enamel demineralization [37]. As related in Gomez et al. [38] both microhardness and QLF methods were able to distinguish in vitro remineralization models using different fluoridated toothpastes. The differences between the initial and post treatment QLF measurements were observed in all experimental groups with the significant recovery of fluorescence and reduction of the lesion area. Despite the statistical equivalence when the means were compared, a higher microhardness means after pH cycling was observed when the specimens were treated with RDC (112.5) (Table 2). The high fluorescence recovery mean presented by the RDC demonstrated a greater mineral gain (ΔF_RE_) in the specimens treated with the REFIX-containing toothpaste (Fig. 1).

Reasons that explain the better results for the REFIX-containing toothpaste rely on its effects on the surface morphology of the specimens after treatment. The SEM micrographs show a mineralized layer formed on the enamel surface after pH cycling interspersed with the REFIX toothpaste treatment (Fig. 2c). A previous study demonstrated that the formation of a silicon-enriched mineral layer on the enamel surface induced by the REFIX-based toothpaste was favored by the formation of complexes of the bioactive particles of calcium, phosphorus, and sodium [39]. The elemental mapping analysis of RDC also corroborated the presence of silicon (7.14%) in the formulation (Table 3). Substituting phosphate groups with silicon affects the mechanical properties of the silicon-enriched hydroxyapatite [40]. When associated with fluorine and phosphate groups, the silicon content enhanced the bioactivity and apatite-forming ability of hydroxyapatite, substituting with silicon in to the remineralizing hydroxyapatite [14, 15]. These findings were corroborated in previous studies [21, 39], that also demonstrated the formation of a mineral layer rich in calcium and silicon in the specimens treated with this same toothpaste.

Like RDC, the anti-erosive toothpaste PC, which contains tetrasodium pyrophosphate associated with sodium fluoride, was found to be as effective as RDC at remineralizing the enamel according to the %SMH_R_ analysis. PC contains the highest content of calcium associated with phosphates, in the form of calcium carbonate, dicalcium phosphate dihydrate, and calcium pyrophosphate [41]. PC also contains a proprietary Pro-Argin technology with 8% arginine, which is indicated against tooth hypersensitivity [42]. This arginine-containing toothpaste has been regarded as offering a potential caries prevention [43]. Unlike RDC, no mineralized layer on the enamel surface was observed in the microscopy analysis (Fig. 2b).

RES contains NR-5 technology, which combines calcium silicate, sodium phosphate salts, and fluoride, seems to improve the remineralization of hydroxyapatite by the nucleation of minerals in tooth enamel in the presence of saliva [44]. SRP contains NOVAMIN technology which comprises an amorphous inorganic of sodium and calcium phosphosilicate [45]. According to the technical profile, serial chemical reactions occurs when bioactive glass is in contact with an aqueous solution, leading to the formation of an insoluble mineralized carbonated hydroxyapatite layer on the surface of the dentin tissue. Conversely, in enamel, this technology favors another mechanism of action, which seems to reinforce the structure of the enamel hydroxyapatite rather than forming a superficial mineralized layer on the tissue. Despite the claim of the formation of a less-soluble surface hydroxyapatite that is resistant to acid challenges [46], the presence of a mineralized layer on the enamel surface was not confirmed in microscopic analysis (Fig. 2).

The difference in the outcomes of the present study support speculation that changes in the hydroxyapatite structure may occur due to treatment of enamel with the fluoride toothpastes associated with different technologies. Depending on the substitution at the different sites of the hydroxyapatite structure, changes in calcium phosphates nucleation, hydroxyapatite growth and crystallization thermodynamics and kinetics, and ultimately its stability may occur in an oral environment rich in ions released by the toothpastes [47]. In this manner, changes in the mineral phase of teeth may to occur in terms of stoichiometric hydroxyapatite. Unfortunately, the mineral phase of teeth has been erroneously attributed to the stoichiometry of hydroxyapatite of 1.67 [9]. In fact, biological apatite usually comprises a non-stoichiometric hydroxyapatite, being calcium deficient (Ca/P < 1.67), with small crystals and poor crystallinity, leading to a relatively high solubility [9, 48]. Except for PC, which exhibited the lowest Ca/P ratio (1.58), the silicon-rich fluoride toothpastes RDC, RES, and SRP presented the highest ratios, higher than 1.86 (Table 4). Variability in the Ca/P ratio may explain the different results regarding hardness and mineral quantification (QLF). Thus, the research hypothesis, which anticipated that the remineralization of the enamel would be positively affected by the use of fluoride toothpastes after pH cycling, irrespective of their technology, was accepted.

Despite the limitations of this in vitro methodology, every effort was made to simulate the variables in the oral environment, specifically the pH cycle in which the demineralization and remineralization processes that occur in situ or in vivo analyses are intercalated with toothpaste exposure [49]. These analyses allowed a complex control of conditions at a reduced cost to test the efficacy of products designated to remineralize the enamel tissue. It is important to mention that the positive control (PC) used in this study did not only contain sodium fluoride but also had arginine, tetrasodium pyrophosphate, and triclosan as bioactive ingredients. This may have influenced the good performance of this toothpaste, highlighting the suggestion for further studies using only a fluoride toothpaste as a control, without the addition of other compounds.

Although this study did not use cross-sectional microhardness, different analyses were performed on the specimens, such as surface microhardness. This provides data on surface mineral deposition and surface hardening, suggesting a pattern of remineralization and recovery. In addition, the QLF is a validated method for verifying the remineralization of caries lesions in vivo, and can be extrapolated to in vitro studies. The analyses with SEM and EDS also allowed us to identify the formation of a mineral layer containing silicon, suggesting a remineralizing potential of the RDC, which was the initial objective of this study [26, 28, 38, 39, 50, 51].

## Conclusion

The present study demonstrated that all fluoride-containing toothpastes showed remineralizing potential for demineralized enamel associated with pH cycling, compared to toothpaste without fluoride. The toothpaste containing the REFIX technology had the highest mean surface hardness and light-induced fluorescence recovery from carious lesions. The anti-erosive toothpaste containing arginine and tetrasodium pyrophosphate also proportioned surface mineral deposition according to surface microhardness recovery analysis. On the other hand, only the REFIX technology was able to promote the formation of a silicon-rich mineralized layer on the enamel surface.

## Data Availability

All the data generated or analyzed during this study are included in this published article.

## Abbreviations

SH_0_: Baseline enamel Vickers surface microhardness
VHN: Vickers hardness
NC: Negative control (Fluoride-free toothpaste Colgate)
PC: Positive control (Colgate total 12)
SH_1_: Post-demineralization surface hardness
SH_2_: After treatment surface hardness
%SMH_R_: Surface microhardness recovery
QLF: Quantitative light-induced fluorescence
ΔF value: Percentage decrease in the auto fluorescence intensity and changes in the mineral contents of enamel
ΔF_0_: A loss of initial fluorescence, passing through the difference between the sound and demineralized enamel
ΔF_1_: Difference in final fluorescence, using the difference between the sound enamel and the area treated with the toothpastes
ΔF_RE_: Percentage of fluorescence recovery
SEM: Scanning electronic microscopy
EDS: Energy-dispersive X-ray spectroscopy
kV: Kilovolts
ANOVA: One-way analysis of variance
RDC: Regenerador + sensitive dentalclean
RES: Regenerate enamel science
SRP: Sensodyne repair and protect
